# Transgenic Rescue of the LARGE_myd_ Mouse: A LARGE Therapeutic Window?

**DOI:** 10.1371/journal.pone.0159853

**Published:** 2016-07-28

**Authors:** J. C. W. Hildyard, E. Lacey, H. Booler, M. Hopkinson, D. J. Wells, S. C. Brown

**Affiliations:** Department of Comparative Biomedical Sciences, Royal Veterinary College, London NW1 0TU, United Kingdom; University of Minnesota Medical Center, UNITED STATES

## Abstract

LARGE is a glycosyltransferase involved in glycosylation of α-dystroglycan (α-DG). Absence of this protein in the LARGE_myd_ mouse results in α-DG hypoglycosylation, and is associated with central nervous system abnormalities and progressive muscular dystrophy. Up-regulation of LARGE has previously been proposed as a therapy for the secondary dystroglycanopathies: overexpression in cells compensates for defects in multiple dystroglycanopathy genes. Counterintuitively, LARGE overexpression in an FKRP-deficient mouse *exacerbates* pathology, suggesting that modulation of α-DG glycosylation requires further investigation. Here we demonstrate that transgenic expression of human LARGE (LARGE-LV5) in the LARGE_myd_ mouse restores α-DG glycosylation (with marked hyperglycosylation in muscle) and that this corrects both the muscle pathology and brain architecture. By quantitative analyses of LARGE transcripts we also here show that levels of transgenic and endogenous LARGE in the brains of transgenic animals are comparable, but that the transgene is markedly overexpressed in heart and particularly skeletal muscle (20–100 fold over endogenous). Our data suggest LARGE overexpression may only be deleterious under a forced regenerative context, such as that resulting from a reduction in FKRP: in the absence of such a defect we show that systemic expression of LARGE can indeed act therapeutically, and that even dramatic LARGE overexpression is well-tolerated in heart and skeletal muscle. Moreover, correction of LARGE_myd_ brain pathology with only moderate, near-physiological LARGE expression suggests a generous therapeutic window.

## Introduction

Dystroglycan was originally identified as the central component of the dystrophin associated glycoprotein complex (DAGC) in skeletal muscle, but has since been shown to be one of the main receptors linking basement membranes to the cell surface in a wide variety of tissues, via association with components such as laminin [1], perlecan, agrin [2] in muscle, neurexin in the brain [3], pikachurin in the eye [4] and most recently Slit [5]. Dystroglycan consequently plays a primary role in the deposition, organisation and turnover of these specialised matrices, mediating basement membrane formation [6, 7], synaptic plasticity [8, 9], neuronal cytoskeletal remodelling [10, 11], axon guidance [5, 12], three-dimensional organisation of radial glia [13], cell adhesion [14], and acting as a scaffold to facilitate localisation of signalling molecules close to their sites of action [15].

Dystroglycan is comprised of two subunits, α- and β-DG; both products of a single gene (*Dag1*), translated as a single protein and post-translationally cleaved. Whilst β-dystroglycan is recognised as a transmembrane adhesion protein with many interacting partners [16, 17], α-DG is a heavily-glycosylated peripheral membrane protein that consists of two globular domains connected by an elongated central domain [18]. This central domain is extensively O-mannosylated on Ser/Thr residues, and glycoepitopes in this region contribute to the stability of the protein but also govern linkage to laminin-G-like (LG) domain-containing extracellular matrix proteins [2, 19], with binding strength and specificity mediated both by the nature and extent of glycosylation.

Defects in glycosylation of α-DG are thus central to the disease process in a group of disorders now referred to as the secondary dystroglycanopathies. Mutations in up to 18 genes have now been associated with this group, including *POMT1* [20], *POMT2* [21], *POMGnT1* [22, 23], *Large* [12, 24], *FKTN* [10, 25, 26], *FKRP* [27], *DPM1*, *DPM2* [28, 29], *ISPD* [9, 30, 31], *GTDC2* [32], *TMEM5* [9], *B3GNT1* [33], *DOLK* [34], *SGK196* [35], *GMPPA* and *GMPPB* [36, 37]. Mutations in these genes give rise to a wide spectrum of clinical phenotypes of varying severity: Walker-Warburg syndrome and Muscle-Eye-Brain disease are invariably fatal, while mutations leading to congenital muscular dystrophies (CMD) can affect muscle alone, or present with ocular and central nervous system defects (including cortical malformations such as polymicrogyria and cobblestone lissencephaly). Even limb-girdle muscular dystrophies (LGMD) can vary in disease progression and severity. With respect to *Large* (like-acetylglucosaminyltransferase), to date 15 patients with mutations in this gene have been reported and whilst these patients display a wide clinical phenotype they all present with brain involvement [38].

Post-translational addition of the polysaccharide repeating unit [-3-xylose-α1,3-glucuronic acid-β1-]n by the dual-function LARGE glycosyltransferase both increases and enhances the binding capacity of α-DG for extracellular matrix ligand [39], and some years ago LARGE was shown to be able to compensate for an absence or reduction of POMT1, POMGnT1, fukutin or FKRP although subsequent work showed that this depended on presence of residual α-DG O-mannosylation [40]. This stimulated much interest in the value of LARGE up-regulation as a therapeutic agent in these disorders and promisingly it was shown that its over-expression in wild type mice seemed to cause only a sub-clinical phenotype in older mice [41]. However, overexpressing LARGE in a FKRP deficient model unexpectedly resulted in a *worsening* of the muscle pathology [42], and similar outcomes were observed with LARGE expression in laminin α2 deficient (dy/dy) mice and conditional knock-outs for fukutin [43–45]. A greater understanding of the mechanisms underlying this exacerbation is critical to the development of effective future therapies using LARGE or other agents to hyperglycosylate α-DG.

In this study we sought to determine whether the deleterious phenotype we previously reported following LARGE overexpression in FKRP-deficient muscle was a sufficiently-generalised phenomenon such that it would manifest even under conditions where deficiency in LARGE represented the sole defect. In order to do this we crossed the same LARGE transgenic line [41] as used in the previous study [42] with the LARGE_myd_ mouse which carries a deletion of exons 5–7 of the *Large* gene, resulting in frameshift and a concomitant premature termination codon (PTC) [24]. LARGE_myd_ mice display a muscular dystrophy phenotype in addition to both cortical and cerebellar abnormalities [12, 24], presenting at the phenotypic level as reduced lifespan, pronounced kyphosis, muscle atrophy and a characteristic hindlimb paralysis.

We show here that transgenic expression of human LARGE on the LARGE_myd_ background restores a normal phenotype and wholly corrects both the muscle pathology and the abnormalities seen in the brain, despite dramatic tissue-specific differences in transgene expression level and extent of α-DG hyperglycosylation. Our data suggests LARGE-mediated hyperglycosylation of muscle α-DG is well-tolerated, and may indeed only be deleterious under conditions of continuous muscle regeneration (i.e those caused by additional glycosylation defects such as a loss/reduction of FKRP activity). We further show, however, that this non-physiological hyperglycosylation is not necessarily *required* for functional recovery. This work has important implications for future therapeutic strategies, suggesting LARGE dosing is highly permissive, and that the crucial factor determining the efficacy of LARGE up-regulation in the dystroglycanopathies is the primary gene defect rather than the level of transgene expression.

## Materials and Methods

All reagents were supplied by Sigma unless stated otherwise.

### Mice

All mice used for this study (Wild type, LARGE_myd_, LARGE_myd_-LV5 and WT-LV5 genotypes -LV5 suffixes indicating presence of the human LARGE transgene [41]) are on a predominantly C57BL6/J background (5–7 back-crossings). Animal experiments were carried out under license from the Home Office (UK) in accordance with The Animals (Scientific Procedures) Act 1986 and were approved by the Royal Veterinary College ethics and welfare committee.

A total of 52 mice were used for this study: 10–15 mice per genotype in approximately equal ratios of males and females, collected at 10–22 weeks of age. No sex or age-dependent differences were observed in any genotype in the relevant parameters measured.

#### Sample preparation

Mice were killed by cervical dislocation and tissues harvested rapidly. Tissues for protein/RNA analysis were flash-frozen in liquid nitrogen. For histology skeletal muscle was mounted in cryo-M-bed (Bright) on cork blocks and frozen under liquid nitrogen-cooled isopentane. In order to preserve tissue morphology brain tissue was fixed in paraformaldehyde, dehydrated and paraffin wax-embedded. Samples were serial sectioned at 10 μm (frozen muscle) and 5μm (paraffin wax embedded brain), with sections collected onto charged slides (Superfrost Plus, VWR). Haematoxylin and eosin staining was carried out using standard protocols.

### Histology

#### Brains

Coronal sections of brains were examined at five levels–the cerebral cortex at the levels of the optic chiasm and the posterior hypothalamus, the pons, the midcerebellum and the medulla oblongata. Sections were deparaffinised, rehydrated, and either stained with haematoxylin and eosin, or taken through heat-induced epitope retrieval (HIER) for immunohistochemistry using antibodies to glycosylated α-DG (IIH6, Millipore) and to β-DG (Clone 56, BD biosciences). HIER was performed in Tris-EDTA (10 mM Tris-HCl, 1 mM disodium EDTA, pH 8.0). Primary antibodies were diluted in phosphate buffered saline containing 0.05% tween 20 and 5% goat serum to 1:200 (IIH6) or 1:100 (βDG) and incubated on sections for 1 hour at room temperature. Visualisation of antibody binding used Envision (DAKO).

#### Skeletal Muscle

Sections of quadriceps muscles were either stained with haematoxylin and eosin or immunostained using IIH6 and β-DG antibodies (as above) with AlexaFluor (488 and 594) conjugated secondary antibodies. Nuclei were resolved using Hoechst staining. Photomicrographs of colorimetric-stained sections were captured on a Leica DM4000B brightfield microscope using a Leica DC500 camera. Immunofluorescent images were captured on a Leica DM4000 fluorescence microscope using a monochrome camera.

### Preparation of tissue lysates

Flash-frozen tissues (whole brains, quadriceps muscle) from individual mice were pulverised under liquid nitrogen using a mortar and pestle, and subsequent powdered tissue (ca. 50-100mg per sample) was added directly to lysis buffer containing 1% (vol/vol) Triton X-100, 0.1% (wt/vol) SDS supplemented with Complete mini Protease Inhibitors (Roche). Samples were incubated on ice for 1 hour then boiled for 5 min. Insoluble material was removed by centrifugation (13,000 rpm for 15 min at 4°). Lysate protein levels were measured using the BCA kit (Pierce), and stored at -80 for use in western blotting.

### Western Blotting

Tissue lysates (10-25ug.lane-1) were run on precast acrylamide gradient gels (3–8%, Invitrogen) and transferred to PVDF membranes using wet blotting. Blots were blocked for 1 hour in 5% milk (Marvel) in TBS-T (TBS supplemented with 0.5% Tween 20) before probing with IIH6 (Millipore) at 1/100 in 5% milk TBS-T, followed by HRP-conjugated anti-mouse IgM (1/100000). Blots were developed using ECL prime and visualised using Hyperfilm (Amersham) or a ChemiDoc MP imaging system (BioRad).

### Cell culture

H^2K^SF1 and H^2K^2B4 (*mdx* and WT immortomouse-derived cell lines, respectively) were grown in matrigel-coated flasks (0.1mg.ml^-1^) and maintained in a proliferative state by incubation at 33°C in proliferation medium: DMEM+glutamax (invitrogen) supplemented with 20% (v/v) heat inactivated-foetal bovine serum, 2% (v/v) chicken embryo extract (CEE, Sera laboratories international), 1% (v/v) Penicillin/Streptomycin (Sigma, final concentration 100u.ml^-1^ penicillin, 100ug.ml^-1^ streptomycin), and 20 U/mL γ-IFN (Chemicon). One day prior to differentiation, cells were seeded onto matrigel-coated 6-well plates at 2x10^5^ cells.well^-1^ (2B4) and 5x10^5^ cells.well^-1^ (SF1), plating conditions established to allow optimal differentiation without under- or overcrowding cells. Differentiation was initiated by replacement of growth media with differentiation medium (DMEM+glutamax supplemented with 5% horse serum (PAA) and 1% pen/strep) and incubation at 37°C. Differentiation medium was partially replaced (50% of medium aspirated, replaced with fresh differentiation medium) after 5 days of differentiation.

### RNA isolation and cDNA synthesis

Flash-frozen tissues (brains, hearts, quadriceps muscle) were pulverised under liquid nitrogen using a mortar and pestle, and subsequent powdered tissue (ca. 100mg per sample) was used to prepare RNA using TRIzol reagent. Myoblast/myotube cell cultures were harvested by cell scraper into TRIzol directly. RNA was prepared according to the manufacturer’s protocol with an additional chloroform extraction step (1:1 vol/vol) added prior to precipitation. Samples were analysed via denaturing agarose gel for presence of two sharp ribosomal bands, and RNA quality was measured via nanodrop spectrophotometry to ensure 260/230 ratios higher than 1.8 (typically > 2.0), and 260/280 ratios of 1.9–2.0. Samples with a 260/230 ratio lower than 1.8 were cleaned via an additional ethanol precipitation (0.1 vols 3M NaAc pH 5.5, 10ug glycogen, 3 vols ice cold ethanol, -80 degrees for 1 hour).

cDNA was prepared from 0.8-1ug of RNA, using the RTnanoscript kit (PrimerDesign) with oligo dT and random priming.

### Quantitative PCR

Primers for endogenous LARGE (mLarge), the LARGE LV5 transgene (hLarge) and endogenous LARGE2 (mLarge2) were designed using Primer3 (primer3.ut.ee). PCR products were typically 80-200bp in length, and designed to span one or more introns (where possible) to prevent amplification of genomic DNA. Primer sequences were selected to minimise cross-reactivity, as mouse LARGE, human LARGE and mouse LARGE2 transcripts share a high degree of sequence identity. Several primer sets were designed for LARGE2 (see S1 Sequences), though we did not detect LARGE2 transcript in any tissues described here. Primers to Csnk2a2, Pak1ip1, Htatsf1, Ap3d1 and Zfp91 form part of the geNorm^Plus^ kit, and are proprietary property of PrimerDesign.

mLarge exon14 F1: CTCACATTCATGGAATTGGATGCA

mLarge exon15 R1: TGGTGGCTCTCCCTAGTAGT

hLarge 5UTR F1: GAGCTCGGATCCACTAGTCC

hLarge 5UTR R1: CGAAGCTCCCAGAAAACAGG

qPCR runs used Precision SYBR green mastermix (Primerdesign) in white hard-shell 384-well plates (BioRad) using 5-10ng of cDNA per well in a BioRad CFX384. Cq values for LARGE and LARGE LV5 were converted to relative quantities (or transcript numbers as described below) and normalised to the geometric mean of two or three suitable reference genes (as determined by geNorm and Normfinder): Cell culture: Cnsk2a2 and Ap3d1, Heart and quadriceps: Pak1ip1 and Ap3d1, Brain: Pak1ip1, Htatsf1 and Zfp91. Mean expression levels per tissue of the shared reference gene (Pak1ip1) were used to calibrate normalisation factors to determine relative transcript levels across tissues. Cq values were typically between 20 and 27 for both reference genes and genes of interest (though as low as 17 for LARGE LV5 in heart/quadriceps).

Estimations of transcript numbers were performed by qPCR using constant amounts of sample cDNAs (or water) mixed with a standard dilution series (10^7^−10^0^ molecules.well^-1^) prepared from purified PCR product of known concentration. As cDNA is predominantly single-stranded an additional cycle is required for parity with PCR products, thus the concentration (in molecules.well^-1^ of standard) at which the dilution series (+cDNA) plateaus corresponds to approximately half the cDNA concentration.

### Statistics

P values in figure legends refer to statistical calculations performed using multiple comparisons tests (Dunn’s or Holm-Sidak’s as appropriate with respect to data normality and N value).

## Results

### Transgenic overexpression of LARGE restores glycosylation of α-DG in both muscle and brain of LARGE_myd_ mice, and corrects both the muscle and brain defects

Wild type mice carrying the LARGE-LV5 transgene (WT-LV5) are indistinguishable from non-transgenic littermates [41], while homozygous LARGE_myd_ mice are readily identified by their smaller size, pronounced kyphosis and distinctive gait abnormalities [12]. LARGE_myd_ mice carrying the LARGE-LV5 transgene (LARGE_myd_-LV5), generated for this study, appear wholly normalized, being essentially identical in appearance to WT or heterozygote littermates.

**Fig 1:**
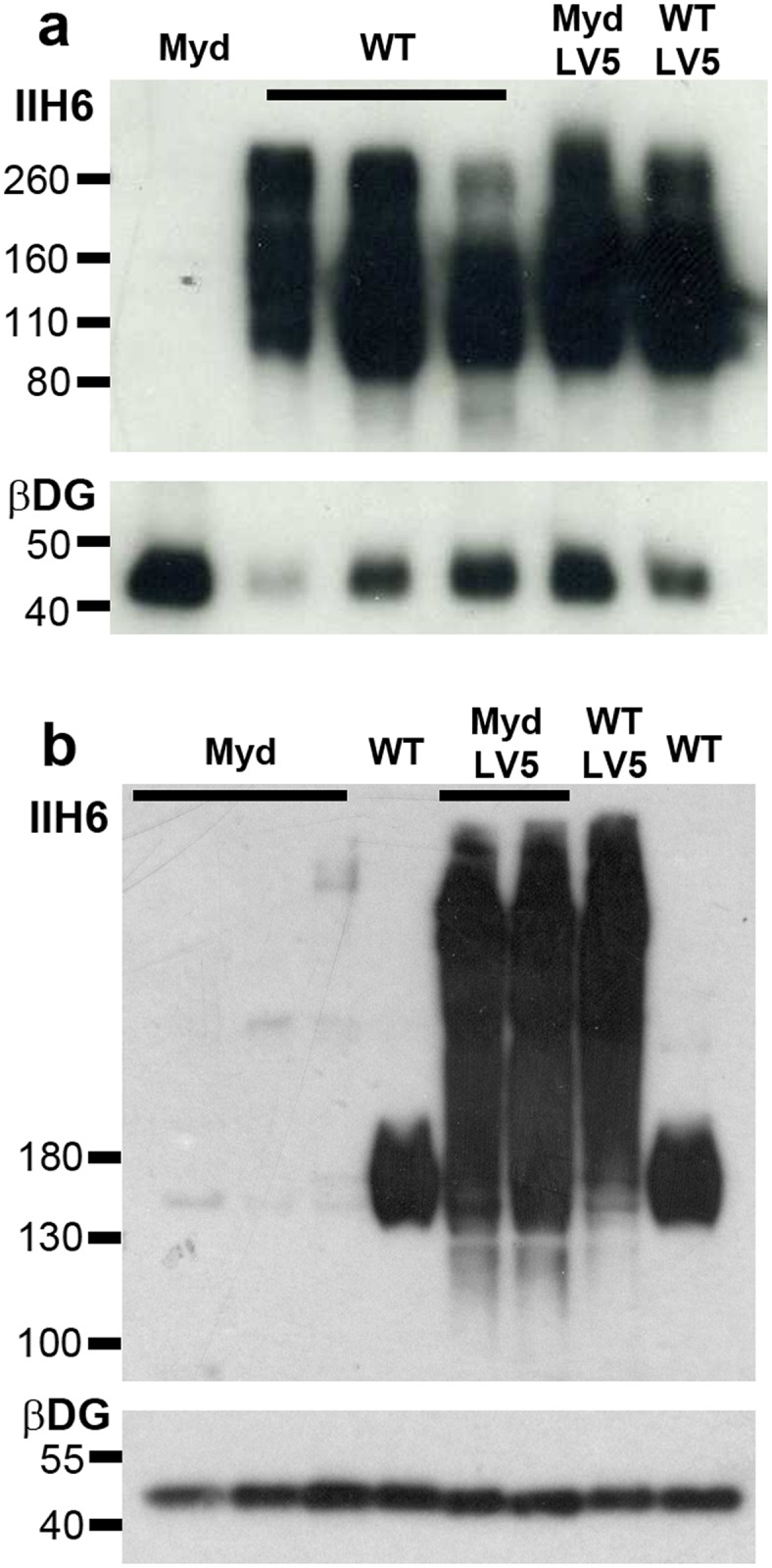
The LARGE-LV5 transgene restores IIH6 reactivity, and causes marked hyperglycosylation in muscle. IIH6 western blot of tissue lysates from brain (a) and quadriceps muscle (b) of WT and LARGE_myd_ mice with or without the LARGE-LV5 transgene (as indicated). β-dystroglycan included as loading control.

Western blotting of tissue lysates prepared from WT, WT-LV5, LARGE_myd_ and LARGE_myd_-LV5 transgenic mice with the antibody IIH6 (which recognizes the laminin-binding epitope of α-dystroglycan) demonstrated restoration of glycosylation on α-DG in LARGE_myd_-LV5 transgenic mice (Fig 1). The muscles of LV5 transgenic LARGE_myd_ mice demonstrated the greatly enhanced IIH6-reactivity characteristic of hyperglycosylation, as did WT-LV5 mice (as previously published [41]). Interestingly the brains of LV5-transgenic WT and LARGE_myd_ mice did not display obvious hyperglycosylation, implying a significant tissue-specific variation in LARGE-LV5 transgene expression.

**Fig 2:**
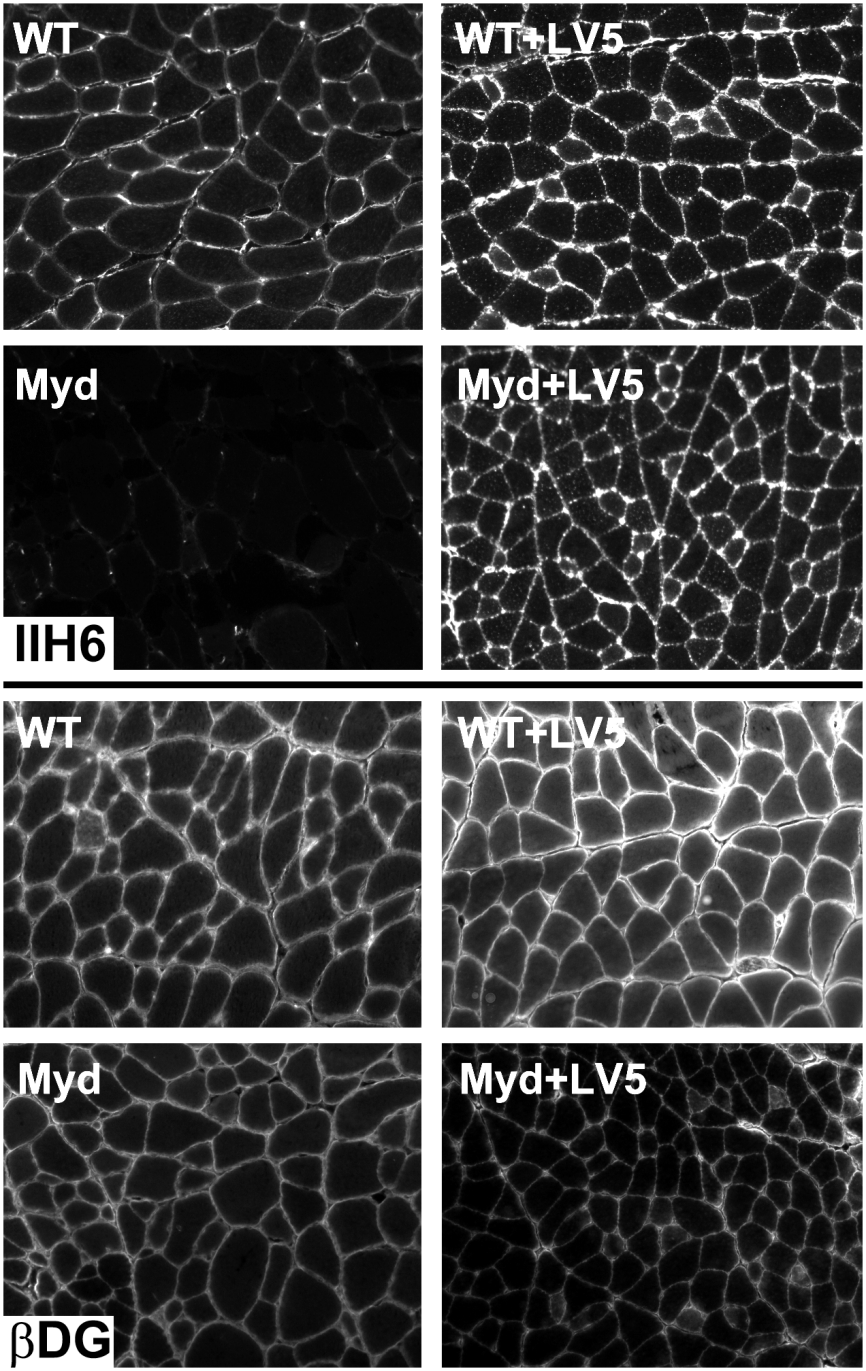
The LARGE-LV5 transgene restores IIH6 reactivity. Immunostaining of quadriceps muscle from WT and LARGE_myd_ mice with or without the LARGE-LV5 transgene (as indicated). Upper panel: IIH6, Lower Panel: β-Dystroglycan.

Skeletal muscle immunohistochemistry confirmed that transgenic expression of LARGE restored IIH6 reactivity in LARGE_myd_ mice, and that the IIH6 epitope was correctly localised to the sarcolemma (Fig 2). Both WT-LV5 and LARGE_myd_-LV5 muscles demonstrated enhanced IIH6 reactivity characteristic of hyperglycosylation.

**Fig 3:**
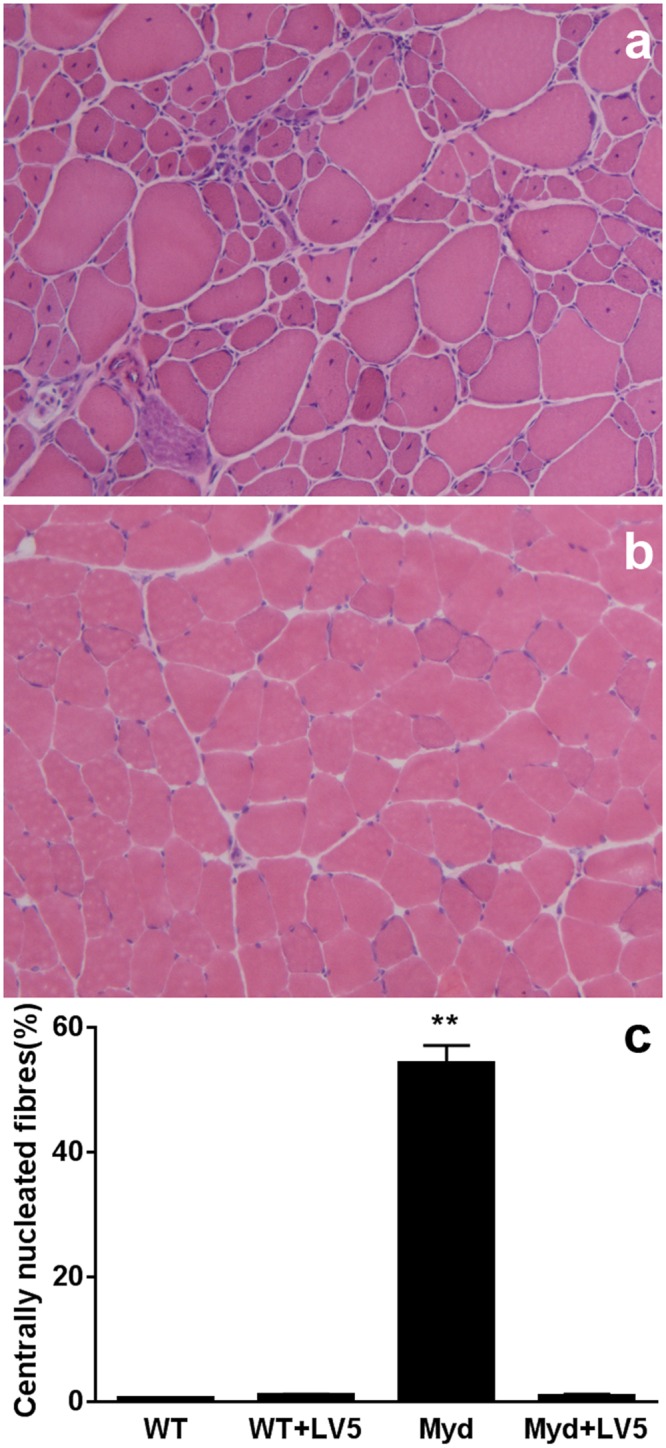
The LARGE-LV5 transgene corrects LARGE_myd_ muscle pathology. Haematoxylin/Eosin staining of quadriceps muscle from LARGE_myd_ (a) and LARGE_myd_ plus the LARGE-LV5 transgene (b). Extent of muscle central nucleation in the genotypes indicated (c). Means+SEM, N = 3. ** P<0.01.

LARGE_myd_ mice typically demonstrate progressive, severe muscle pathology, with extensive fibrotic and adipose infiltrates, increased fibre size variation and large numbers of hypercontractile and centrally nucleated fibres. Muscles from LARGE_myd_-LV5 mice exhibit no visible hypercontractility, infiltration or scarring, and wild-type levels of central nucleation (Fig 3), demonstrating that the muscle phenotype is wholly corrected and further that hyperglycosylation of α-DG appears to have no adverse effects in this tissue.

**Fig 4:**
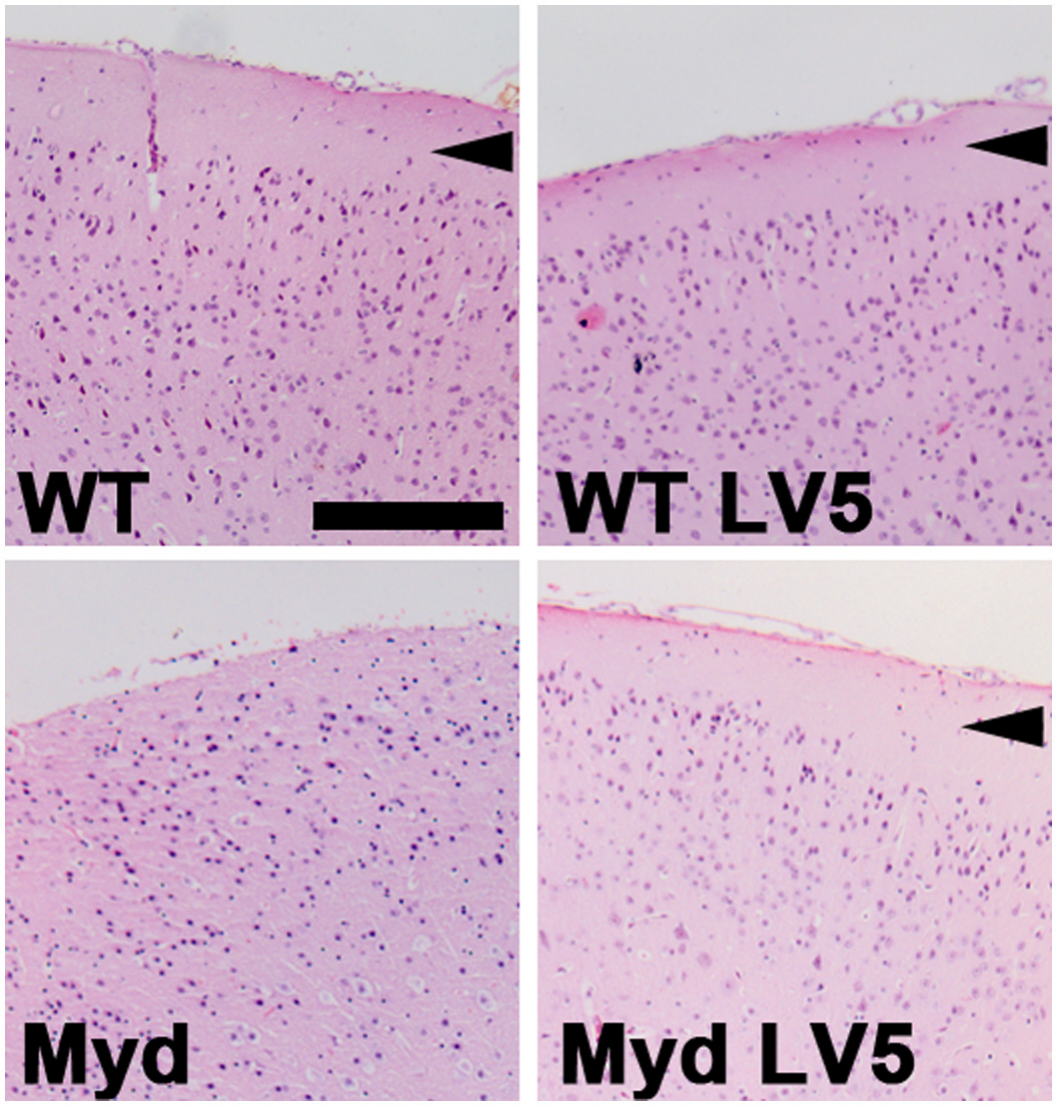
The LARGE-LV5 transgene corrects LARGE_myd_ cortical defects. Haematoxylin/Eosin staining of cortical coronal sections of brains from wild type, WT-LV5, LARGE_myd_ and LARGE_myd_-LV5 mice (as indicated). WT and WT-LV5 mice display normal laminar cortical arrangement, with a clear molecular layer which is lost in LARGE_myd_ but restored by the LARGE-LV5 transgene (arrowheads). Bar: 200μm.

**Fig 5:**
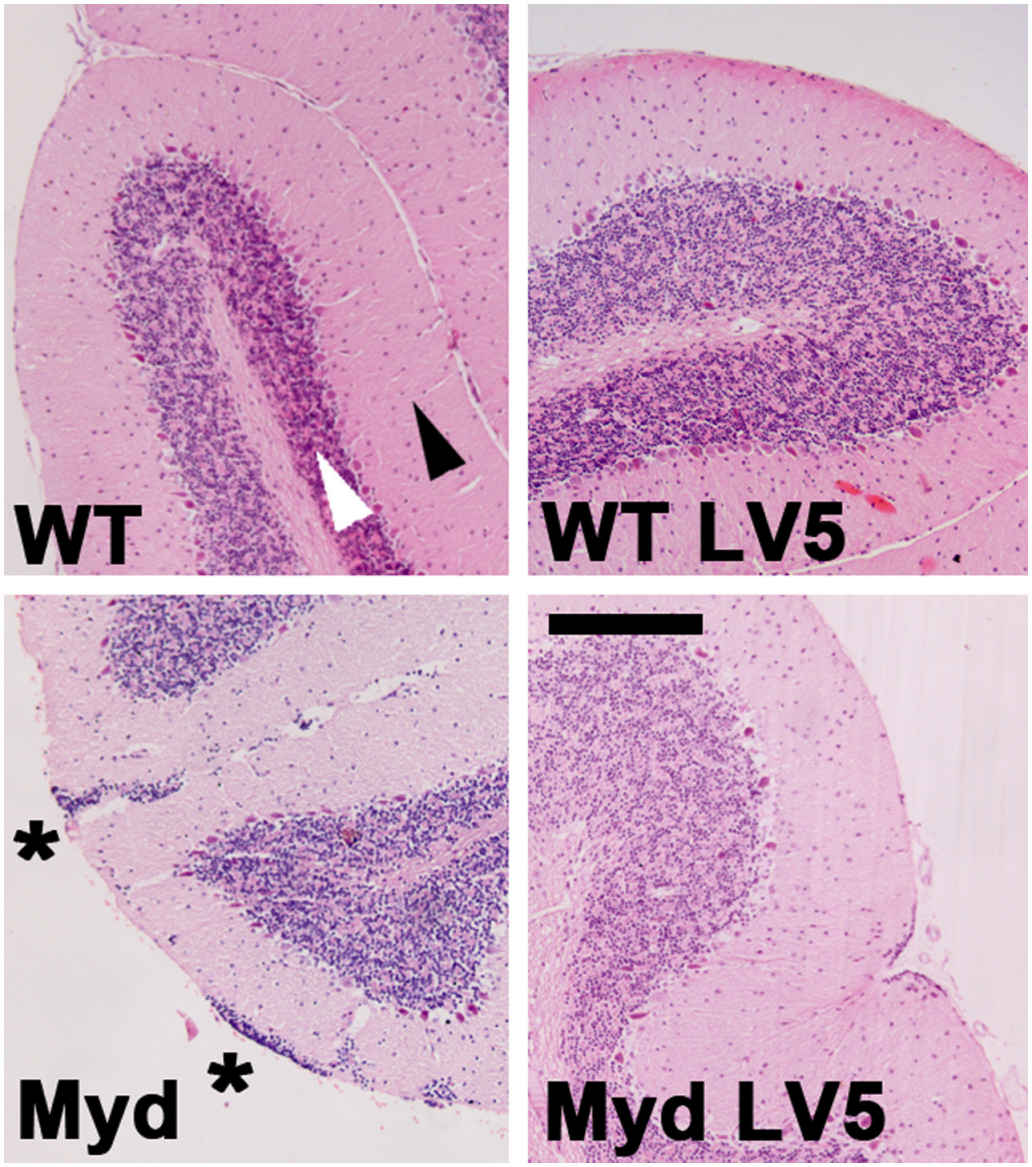
The LARGE-LV5 transgene corrects LARGE_myd_ cerebellar defects. Haematoxylin/Eosin staining of cerebellar sections of brains from wild type, WT-LV5, LARGE_myd_ and LARGE_myd_-LV5 mice (as indicated). The molecular layer (black arrowhead) and granular cell layer (white arrowhead) are readily apparent in WT and WT-LV5 mice, with a single layer of large Purkinjie cells sandwiched between them. In LARGE_myd_ mice, the granular cell layer is extensively disrupted with large aggregates of ectopic granule cells superficial to the molecular layer (asterisks). This disruption is corrected, and ectopic granule foci greatly reduced, in the LARGE_myd_-LV5 mice. Bar: 200μm.

The brains of adult LARGE_myd_ mice typically exhibit lesions characteristic of a neuronal migration defect, with fusion of the interhemispheric fissure and loss of normal cortical lamination. Within the cortex, ectopic neurons infiltrate the marginal zone and form a narrow, multifocal extracortical layer. In addition, normal lamination in the cerebellum is lost, and large clusters of ectopic granule cells are present between the molecular layer and the pia. Haematoxylin and eosin staining (Figs 4 and 5, S1 and S2 Figs) revealed that the brains of both WT-LV5 and LARGE_myd_-LV5 transgenic mice were essentially histologically indistinguishable from WT mice, the only exception being the presence of small focal aggregates of ectopic granule cells (less than 5 cells) that were observed between the molecular layer and the pia within the cerebellum of all LARGE_myd_-LV5 brains but only a single WT brain (n = 6).

**Fig 6:**
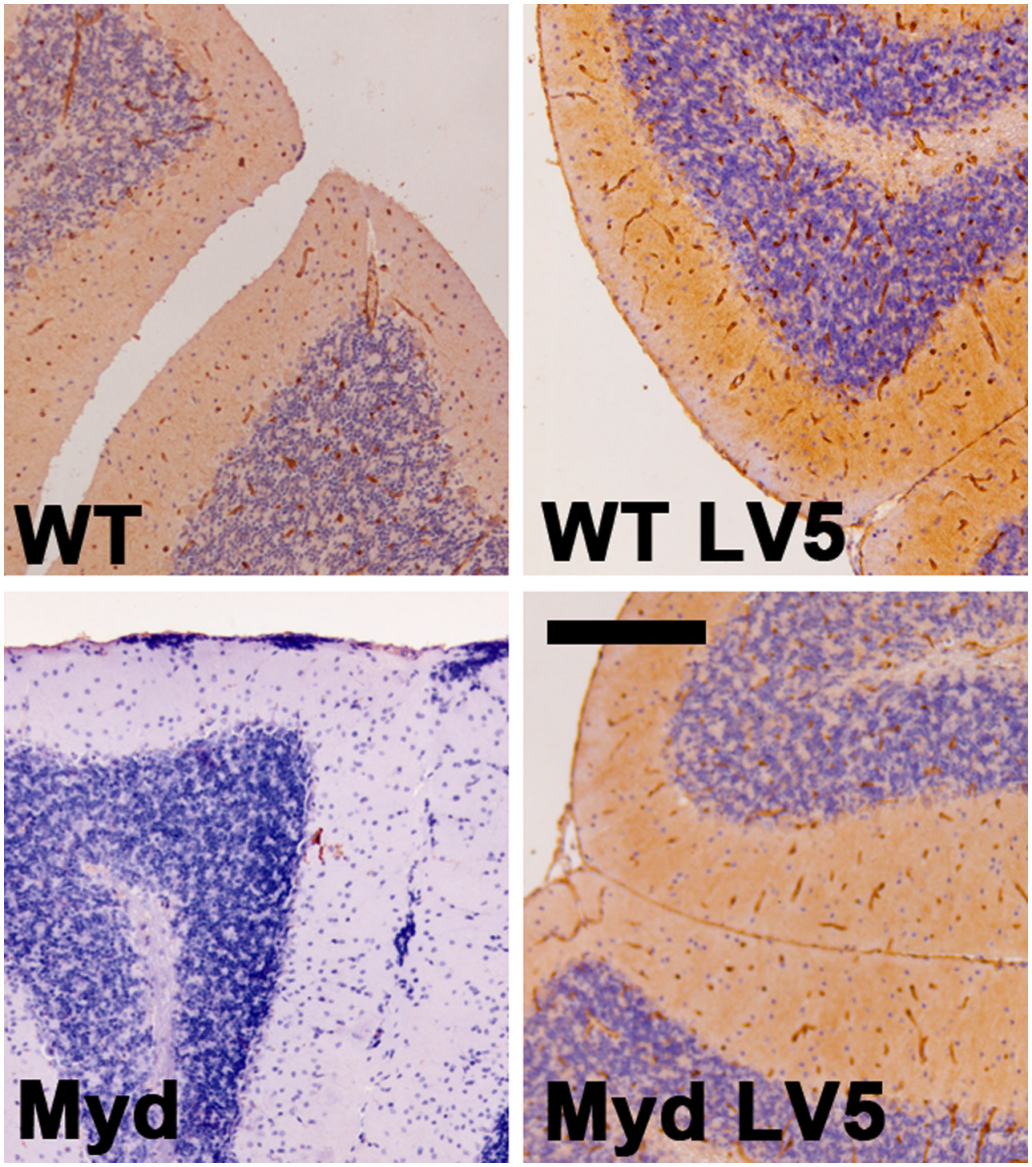
The LARGE-LV5 transgene restores IIH6 reactivity and confers a diffuse global increase in the epitope in brains. IIH6 immunostaining of cerebellar sections of brains from wild type, WT-LV5, LARGE_myd_ and LARGE_myd_-LV5 mice (as indicated). IIH6 reactivity is observed in blood vessels and the pia in WT, WT-LV5 and LARGE_myd_-LV5 cerebellum, but not in brains of LARGE_myd_ mice. Overall stain intensity is higher in LV5 transgenic mice than in WT controls. Bar: 200μm.

IIH6 immunoreactivity in WT brains primarily localises to the vascular and pial basement membranes and the choroid plexus: while choroid plexus staining is retained in LARGE_myd_ mice [8], IIH6 immunoreactivity is absent in all other areas (Fig 6, S1 and S2 Figs). Transgenic expression of LARGE in LARGE_myd_-LV5 brains restored a WT staining pattern, though diffuse global immunoreactivity was also observed in both LARGE_myd_-LV5 and WT-LV5 brains.

Staining for β-DG was of similar pattern and intensity between WT, LARGE_myd_-LV5 and WT-LV5 mice. Interestingly LARGE_myd_ mice exhibited a marked decrease in staining intensity of β-DG specifically at vascular basement membranes, while also exhibiting faint diffuse staining of the cerebellar molecular layer (S1 and S2 Figs). Expression of β-DG at the pial basement membrane and in the epithelium of the choroid plexus was similar to that observed in the other groups. Taken together, histopathological and immunohistochemical findings indicate that expression of the LARGE-LV5 transgene in the LARGE_myd_ mouse rescues the brain phenotype, both structurally and at the protein level.

### Levels of transgenic LARGE transcript are close to physiological values in brain, but dramatically overexpressed in muscle and heart

Previous studies have shown that levels of both endogenous LARGE and the LARGE-LV5 transgene vary significantly from tissue to tissue: endogenous LARGE expression was shown to be high in the brain, relatively low in heart and lower still in skeletal muscle, while in marked contrast expression of the transgene was high in skeletal muscle, low in heart and lower still in brain [41]. These studies, however, only address the relative levels of each transcript in different tissues and do not therefore allow direct comparison of endogenous expression with transgene transcript levels. To determine this relationship precisely we used quantitative PCR (qPCR) to obtain values for absolute number of transcripts in brain, heart and muscle.

**Fig 7:**
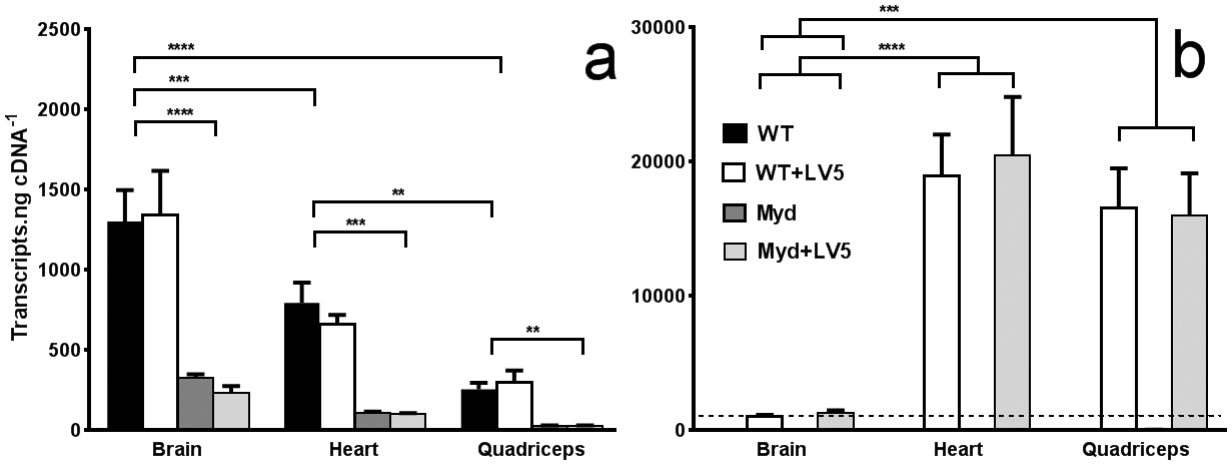
Levels of transgenic LARGE transcript are equivalent to endogenous expression in brain, but greatly overexpressed in heart and skeletal muscle. qPCR analysis of cDNA prepared from brain, heart and quadriceps (from genotypes as indicated) using primers for endogenous LARGE (a) or the LARGE-LV5 transgene (b). Quantities are shown as mean number of transcripts per ng cDNA (+ S.E.M, N = 5–9). Dashed line in (b): expression level of endogenous LARGE in brain. ** P<0.01, *** P<0.005, **** P<0.001. No significant differences in endogenous expression within a given tissue were detected between WT and WT-LV5, or in transgene expression between WT-LV5 and LARGE_myd_-LV5, for any of the tissues studied.

As shown in Fig 7, qPCR analyses confirmed that LARGE transgene expression driven by the CAGGs promoter demonstrates significant tissue specificity, achieving levels of expression in heart and skeletal muscle over an order of magnitude greater than in brain (Fig 7b). Conversely, while levels of endogenous transcript were greatly reduced in LARGE_myd_ mice (as expected for PTC-carrying mRNAs), expression of endogenous LARGE in WT/WT-LV5 mice is 2- or 5-fold higher in the brain than heart or skeletal muscle, respectively (Fig 7a). As a consequence, levels of LARGE expression in the brains of LARGE_myd_ mice carrying the LV5 transgene are comparable with those in WT mice (approx. 1300 transcripts.ng^-1^ cDNA–dashed line, Fig 7b), while transgene expression in the heart and skeletal muscle can reach levels almost 100-fold greater than endogenous muscle equivalents (ca. 20000 vs 200–800). Expression of the LARGE paralog LARGE2 was not detected in either brain or muscle in WT, WT-LV5, LARGE_myd_ or LARGE_myd_-LV5 mice, thus LARGE2 is unlikely to play a role in α-DG glycosylation in either tissue in the adult. The dramatic skeletal muscle overexpression of the LARGE transgene shown here thus likely underpins the hyperglycosylation of α-DG observed in this tissue.

### Expression of endogenous LARGE follows differentiation cues, increasing during myogenesis in both healthy and dystrophic cells

**Fig 8:**
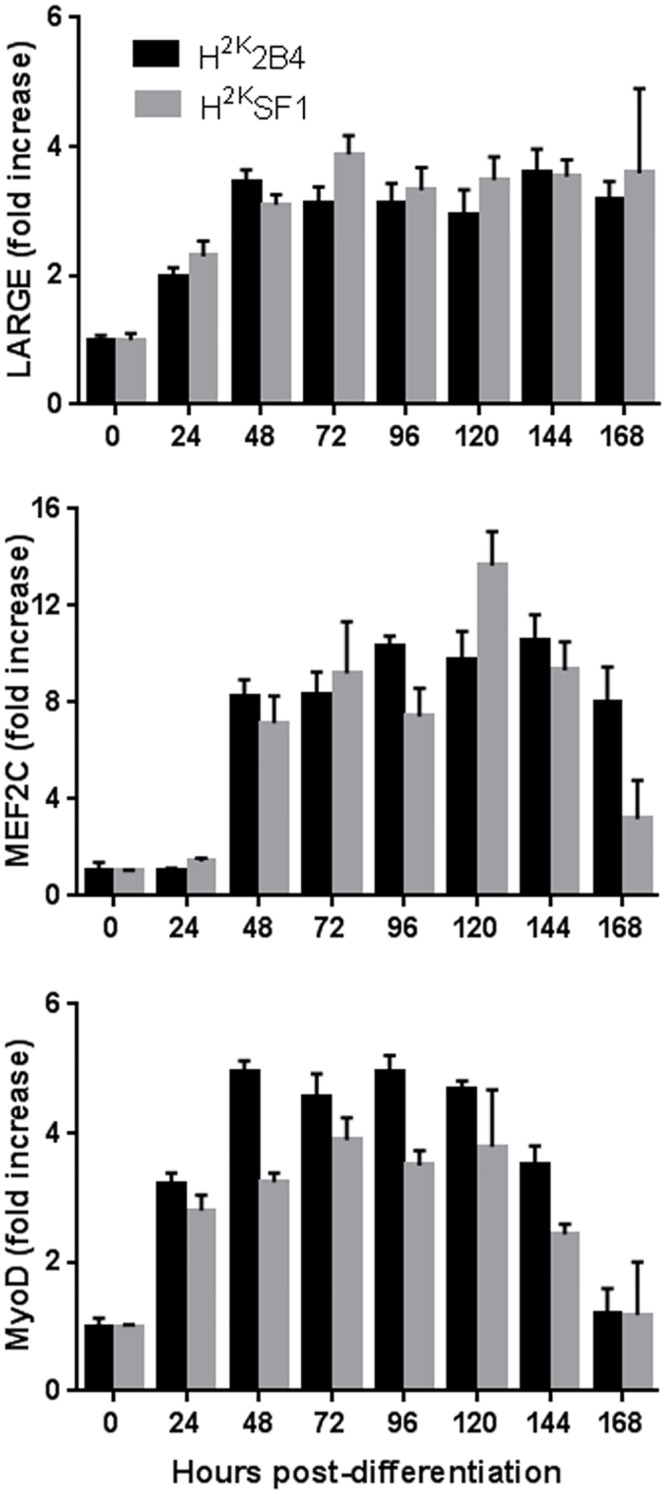
Expression of endogenous LARGE increases during myogenesis in both normal and dystrophic cells. qPCR analysis of cDNA prepared from WT (H^2K^2B4) or *mdx* (H^2K^SF1) cell cultures at the times indicated using primers for endogenous LARGE (upper panel), and for myogenic transcription factors MyoD and MEF2C (lower panels). Expression levels are normalised (using Csnk2a2 + Ap3d1) and shown as fold increase over time-zero (proliferating) levels (Means + S.E.M, N = 3).

Expression of LARGE has been reported to increase during muscle regeneration [39], suggesting a role for this glycosyltransferase in myogenesis. To investigate expression of endogenous LARGE throughout myogenic differentiation under both healthy and dystrophic conditions, we used the immortomouse cell lines H^2K^2B4 and H^2K^SF1, the latter of which carries the *mdx* mutation (the mouse model of Duchenne muscular dystrophy). As shown in Fig 8, expression of LARGE increases over a near-identical time-course in both healthy and dystrophic cultures as cells progress from proliferating myoblasts to large multinucleate myotubes, suggesting expression of LARGE mirrors myogenic progression even in dystrophic myofibres.

## Discussion

Despite promising results in cell culture models of secondary dystroglycanopathies [40], previous work by our group has demonstrated that overexpression of LARGE on a FKRP-deficient background is deleterious [42]. In marked contrast we show here that transgenic overexpression of human LARGE on the LARGE_myd_ background corrects the LARGE_myd_ phenotype in both the skeletal muscle and brain. This is despite a similar pattern of α-DG hyperglycosylation (as judged by both immunocytochemistry and Western blotting) as that observed on the FKRP-deficient background. Relative expression levels of endogenous LARGE and the LARGE-LV5 transgene between tissues are in agreement with previously published data [41], however we also quantified absolute levels of expression and demonstrate that while endogenous and transgene expression levels in brain are highly comparable, heart and skeletal muscle (with their lower endogenous LARGE expression) display a dramatic fold increase in LARGE transcripts, with the transgene expressed at a level almost 2 orders of magnitude greater than endogenous LARGE.

It is important to note that we would not necessarily expect increases in LARGE protein to be of commensurate magnitude with increases in transcript levels in transgenic animals: the LARGE-LV5 transcript does not share 5’ or 3’ UTR sequence with endogenous LARGE transcripts, and these elements (which are of considerable length in the endogenous LARGE mRNA) may influence translation efficiency, stability and subcellular trafficking of the transcript. It is nevertheless reasonable to expect a limited degree of correlation between protein and transcript level, and thus LARGE protein levels are likely to be substantially higher in transgenic muscle than in WT muscle or LV5 transgenic brain tissue.

Our data therefore suggests the α-DG hyperglycosylation observed in transgenic muscle is a consequence of supra-physiological levels of LARGE activity within this tissue (rather than tissue-specific variability in suitable LARGE glycosylation targets) but also suggests that to correct the LARGE_myd_ phenotype in brain, LARGE-LV5 need only be expressed at physiologically-equivalent levels.

Given the nature of our analyses, this observation is somewhat unexpected. The brain is a highly-organised tissue with multiple domains of discrete architecture: in WT brains IIH6 staining is restricted to relatively well-defined regions (primarily the vascular and pial basement membranes and the choroid plexus), implying strict spatio-temporal control. In contrast, expression of the LARGE-LV5 transgene is driven by the relatively potent constitutive CAGGs promoter. Whilst this promoter clearly confers tissue-specific variation in absolute expression level, it is nevertheless likely to be expressing at *some* level, constitutively, in virtually all cells. Immunostaining of LV5 transgenic brains reveals globally elevated IIH6 reactivity (Fig 6, S1 and S2 Figs), indicating that LARGE upregulation is not subdomain-restricted. This is further supported by the observation that LV5 mice even demonstrate IIH6 reactivity in the testis, a tissue usually IIH6-negative (S3 Fig).

Our analysis of gene expression here reflects the total mRNA level in whole adult brain tissue, precluding identification of spatial or temporal variations in gene expression. This is in contrast to muscle, where much of the tissue bulk is comprised of comparatively transcriptionally-uniform myofibres, with specialised regions such as the neuromuscular and myotendinous junctions making only a minor contribution to the transcriptional milieu of a given whole muscle extraction. Measured transcript levels are thus likely a more accurate reflection of homogenous expression in muscle than in brain. With this caveat accepted, the observation that total transgene transcript levels in the brains of LARGE_myd_-LV5 animals are essentially equivalent to those of endogenous LARGE, coupled with the widespread histological IIH6 immunoreactivity LV5-transgenic animals demonstrate, suggests that LARGE expression in LARGE_myd_-LV5 mice does not recapitulate wild-type expression patterns: being low-level and relatively uniform rather than high-level but restricted to specific regions of the tissue.

The phenotypically normal behaviour of WT-LV5 mice suggests that such low-level, widespread and constitutive expression of transgenic LARGE, in the presence of normal expression of endogenous LARGE, does not significantly hinder brain development. The corrected phenotype of LARGE_myd_-LV5 mice, however, strongly suggests that such constitutive expression is in fact *sufficient* to normalise brain development. That low, constitutive expression of LARGE apparently restores normal brain development is thus highly encouraging, if perhaps surprising (though the authors note that vascular IIH6 staining is prominent in WT, WT-LV5 and LARGE_myd_-LV5 brains, thus vasculature-derived LARGE transcripts may comprise a significant percentage of total brain expression levels in both WT and transgenic animals, masking subtle variations).

If LARGE is to function as a therapeutic, elucidation of appropriate dosing is critical. Simply put: how much LARGE is enough? Levels of endogenous LARGE transcript are lower in muscle than brain, and the binding properties of α-DG are different in these two tissues [46]. Taken together, these observations tend to suggest that LARGE may fulfil different (albeit partially overlapping) roles in brain and muscle. As demonstrated here, a 100-fold overexpression of LARGE at the mRNA level is apparently sufficient to rescue the LARGE_myd_ muscle pathology, and moreover with no overt ill-effects. Indeed this same overexpression on a WT background is similarly well-tolerated [41]. As assessed by IIH6 western blotting, this overexpression unsurprisingly leads to hyperglycosylation of α-DG in muscle, while (as might be expected) the near-physiological expression of the transgene in brain apparently does not (Fig 1). Quantitation of this enhanced IIH6-reactivity would be of considerable benefit; however α-DG migrates as a broad, highly diffuse band under SDS-PAGE (an effect yet more pronounced under conditions of hyperglycosylation), making such assessment challenging. These limitations accepted, we feel confident in suggesting that IIH6-reactivity is enhanced by considerably less than 100-fold in LV5-transgenic skeletal muscle. This raises a therapeutically-relevant question: both brains and muscles of WT animals show similar levels of IIH6-reactivity, though as shown here and previously [41] endogenous expression of LARGE mRNA is five-fold higher in brain. Moreover, while a doubling of LARGE transcript appears to have little effect on IIH6-reactivity in brain, a 100-fold increase in LARGE expression leads to a significant but non-commensurate increase in muscle α-DG hyperglycosylation. In essence: why do transcript levels correlate comparatively poorly with IIH6-reactivity?

A simple explanation (as discussed above) is that transcript levels need not correlate with protein levels: a dramatic overexpression at the mRNA level may saturate the translation machinery, or generate more LARGE protein than the Golgi can accommodate leading to degradation. Another possibility lies in the fact that while LARGE is restricted to the Golgi, α-DG resides on the extracellular leaflet of the plasma membrane: the interaction between LARGE and α-DG is subject to spatial and temporal constraints, thus comparatively transient under normal conditions. This interaction may thus be saturable: a finite exposure to the Golgi environment places a limit on potential interaction and glycosylation events, implying a threshold may exist above which additional LARGE protein is unable to contribute to α-DG glycosylation. Conversely, evidence for a physical interaction between LARGE and dystroglycan mediated through the N-terminal domain of α-DG [47] raises the possibility that overexpressed (Golgi-resident) LARGE protein might partially sequester dystroglycan, prolonging DG transit through the Golgi and consequently extending exposure to not only the LARGE glycosyltransferase activity, but also additional Golgi-resident glycosyltransferases.

A further caveat to α-DG-focussed interpretations is that the effects (beneficial or otherwise) of LARGE overexpression need not be wholly mediated through α-DG, or indeed via O-mannosylation: it has been shown that overexpression of LARGE in neural stem cells confers IIH6-reactive LARGE glycan epitopes even in the absence of POMT2 or dystroglycan itself [48]. While at best only an approximation of physiological behaviour, this nevertheless highlights the risks of attributing IIH6-reactivity exclusively to α-DG, and suggests that (in neural tissue at least) multiple targets are capable of accepting LARGE-conferred glycosylation, and not necessarily in a manner dependent on phosphorylated O-mannose. The globally increased IIH6-reactivity observed here in LARGE-LV5 transgenic brains may thus partially reflect non-canonical glycosylation events: the LARGE protein possesses two structurally-distinct glycosyltransferase domains; a degree of promiscuous LARGE activity is not an unrealistic hypothesis.

A critical therapeutic question is why LARGE overexpression (and consequent α-DG hyperglycosylation) so effectively corrects the LARGE_myd_ muscle phenotype, when equivalent LARGE overexpression on a FKRP-deficient background [42] is actively detrimental despite promising behaviour in isolated cells. FKRP-deficient mice carrying the LARGE-LV5 transgene show α-DG hyperglycosylation (suggesting FKRP is not an obligate requirement for addition of LARGE glycans) yet nevertheless exhibit markedly exacerbated disease severity.

Aside from the obvious observation that only LARGE overexpression on a LARGE_myd_ background represents direct complementation, a clue may lie in the observation that LARGE expression increases during myogenic differentiation in both normal and dystrophic cell cultures (Fig 8) and during muscle regeneration *in vivo* [39] and has been proposed to ‘tune’ the extent of interaction with the extracellular matrix environment through α-DG glycosylation. Repair of damaged muscle fibres requires a high degree of coordinated cell proliferation, movement and fusion, and the extracellular matrix of the muscle tissue provides a partial scaffold to organise these processes. While the *absence* of LARGE at any stage leads to α-DG hypoglycosylation and pronounced progressive muscle degeneration, lower (but crucially non-zero) levels of α-DG glycosylation in early differentiation may facilitate myoblast migration and myotube fusion by limiting interaction with matrix components, easing progress through the extracellular environment.

LARGE overexpression (with concomitant α-DG hyperglycosylation) might therefore lead to the reverse, enhancing interaction with the extracellular matrix effectively constitutively, impeding cell movement and hampering regeneration. The phenotypically normal appearance and function of muscle from WT-LV5 or LARGE_myd_-LV5 mice implies that LARGE overexpression does not impair muscle development, thus this effect might be restricted to regeneration alone. We conjecture that detrimental effects of LARGE overexpression may only manifest in the presence of additional muscle defects that necessitate regeneration. WT/LARGE_myd_ mice with the LARGE LV5 transgene (having normal/corrected muscle, respectively) should have wild-type levels of resistance to muscle damage, minimal degeneration and thus limited opportunity to demonstrate a regenerative defect (interestingly, aged WT-LV5 mice do exhibit a mild dystrophic phenotype, possibly representing steady accumulation of poorly-regenerated but sub-clinical muscle damage events [41]). In contrast, FKRP-deficient LARGE-LV5 transgenic mice carry different–uncorrected- defects and thus may still be subject to muscle damage and degeneration (even in the presence of a strengthened interaction between the sarcolemma and the extracellular matrix), allowing the LARGE overexpression-mediated regenerative defect to present, exacerbating disease pathology. Indeed, recently published findings by Saito *et al* [45] support this hypothesis.

In conclusion, the data presented here add support to the viability of LARGE as a potential therapeutic: transgenic expression of LARGE in the LARGE_myd_ mouse wholly corrects both the brain and muscle phenotype in this animal model, despite stark tissue-specific differences in expression level of the transgene. The observation that broadly distributed, low-level expression of LARGE is apparently sufficient to rescue brain morphology suggests substantial overexpression or up-regulation is not necessary for functional restoration, while our muscle analyses demonstrate that dramatic (100-fold) LARGE overexpression–with concomitant α-DG hyperglycosylation- is both sufficient to restore muscle function and also apparently well-tolerated in the absence of additional glycosylation defects (such as the absence of FKRP). Exogenous expression of LARGE would thus appear to exhibit a very broad therapeutic window, providing benefit over a wide expression range. As fine-tuning of gene dosing in gene therapy is non-trivial, the permissive profile described here bolsters the case for the therapeutic use of LARGE.

## Supporting Information

S1 Fig LARGE-LV5 transgene corrects LARGE_myd_ cortical defects. Cortical coronal sections of brains from wild type, WT-LV5, LARGE_myd_ and LARGE_myd_-LV5 mice (as indicated). **a-d**: Haematoxylin/Eosin staining. WT and WT-LV5 mice display normal laminar cortical arrangement (arrowheads), which is lost in LARGE_myd_ but restored by the LARGE-LV5 transgene. **e-h**: IIH6 immunostaining. IIH6 reactivity is observed in blood vessels and the pia in WT, WT-LV5 and LARGE_myd_-LV5 cortex but not in brains of LARGE_myd_ mice. Overall stain intensity is higher in LV5 transgenic mice than in WT controls. **i-l**: β-DG immunostaining. Blood vessels are visible in cortex of WT, WT-LV5 and LARGE_myd_-LV5 cortex but not in brains of LARGE_myd_ mice. Bars represent 200μm. White boxes: image subsections shown in Fig 4 (see main text).
(TIF)

S2 Fig LARGE-LV5 transgene corrects LARGE_myd_ cerebellar defects. Cerebellar sections of brains from wild type, WT-LV5, LARGE_myd_ and LARGE_myd_-LV5 mice (as indicated). **a-d**: Haematoxylin/Eosin staining. In WT and WT-LV5 mice (a, b), the molecular layer (Black arrowhead) and granular cell layer (White arrowhead) are readily apparent, with a single layer of large Purkinjie cells sandwiched between them. In LARGE_myd_ mice (c), the granular cell layer is extensively disrupted with large aggregates of ectopic granule cells superficial to the molecular layer (asterisks). This disruption is corrected, and ectopic granule foci greatly reduced, in the LARGE_myd_-LV5 mice (d). **e-h**: IIH6 immunostaining. IIH6 reactivity is observed in blood vessels and the pia in WT, WT-LV5 and LARGE_myd_-LV5 cerebellum, but not in brains of LARGE_myd_ mice. Overall stain intensity is higher in LV5 transgenic mice than in WT controls. **i-l**: β-DG immunostaining. Pia and blood vessels are visible in the cerebellum of WT, WT-LV5 and LARGE_myd_-LV5 mice but not in LARGE_myd_ mice. LARGE_myd_ mice instead display a diffuse, indistinct staining of the molecular layer. Bars represent 200μm. White boxes: image subsections shown in Figs 5 and 6 (see main text).
(TIF)

S3 Fig LARGE-LV5 transgene confers IIH6 reactivity upon testis. IIH6 western blot of tissue lysates from testis of WT, LARGE_myd_ and LARGE_myd_-LV5 mice (as indicated).
(TIF)

S1 Sequences qPCR primers used for LARGE2. (DOC)
