# Supplementary figures and images for: Transgenic Rescue of the LARGEmyd Mouse: A LARGE Therapeutic Window?
Source: PLoS One. 2016 Jul 28;11(7):e0159853. doi: 10.1371/journal.pone.0159853 (PMC4965172; doi:10.1371/journal.pone.0159853)

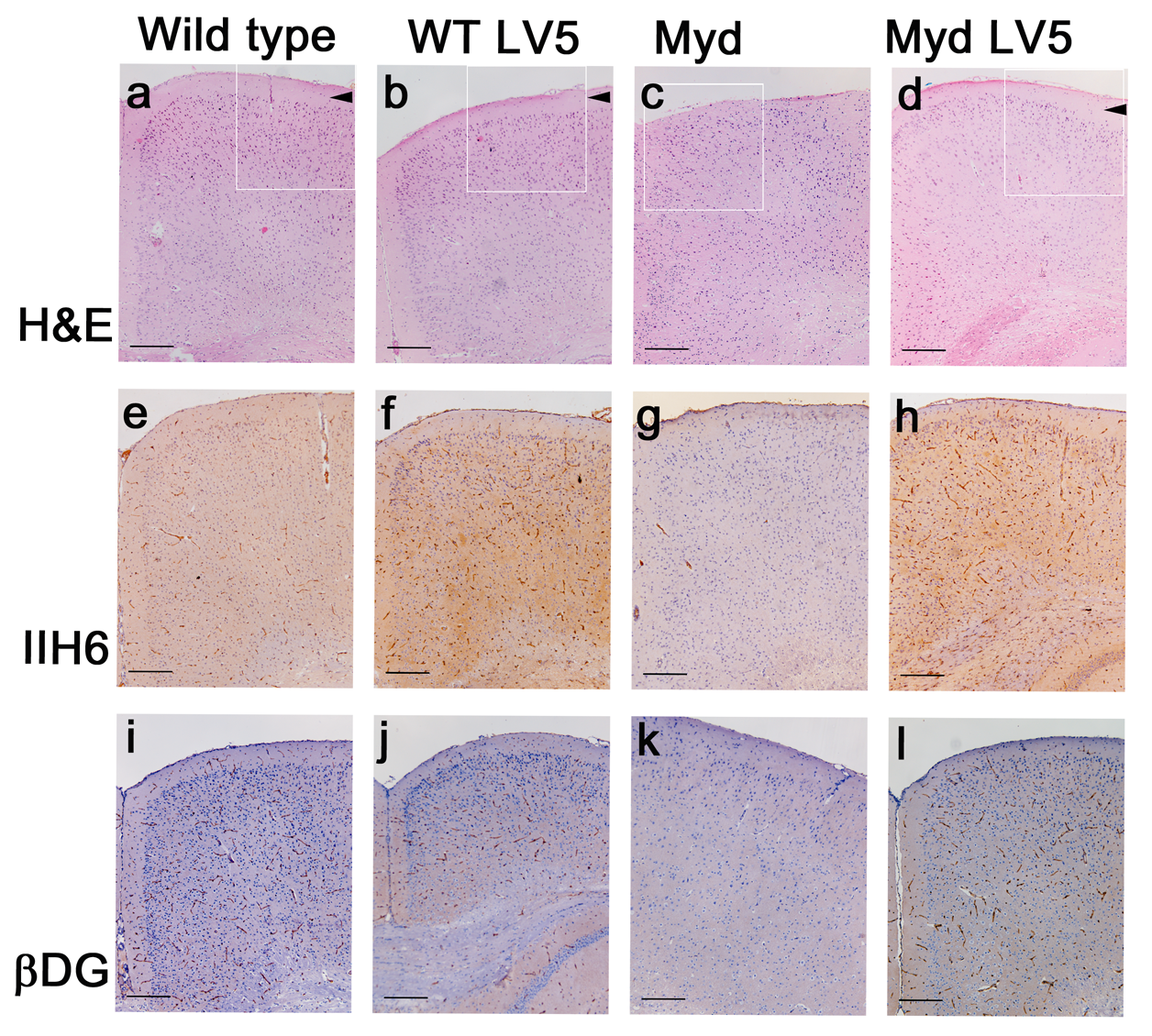

Supplement: S1 Fig — Cortical coronal sections of brains from wild type, WT-LV5, LARGEmyd and LARGEmyd-LV5 mice (as indicated). a-d: Haematoxylin/Eosin staining. WT and WT-LV5 mice display normal laminar cortical arrangement (arrowheads), which is lost in LARGEmyd but restored by the LARGE-LV5 transgene. e-h: IIH6 immunostaining. IIH6 reactivity is observed in blood vessels and the pia in WT, WT-LV5 and LARGEmyd-LV5 cortex but not in brains of LARGEmyd mice. Overall stain intensity is higher in LV5 transgenic mice than in WT controls. i-l: β-DG immunostaining. Blood vessels are visible in cortex of WT, WT-LV5 and LARGEmyd-LV5 cortex but not in brains of LARGEmyd mice. Bars represent 200μm. White boxes: image subsections shown in Fig 4 (see main text). (TIF) [file pone.0159853.s001.tif]

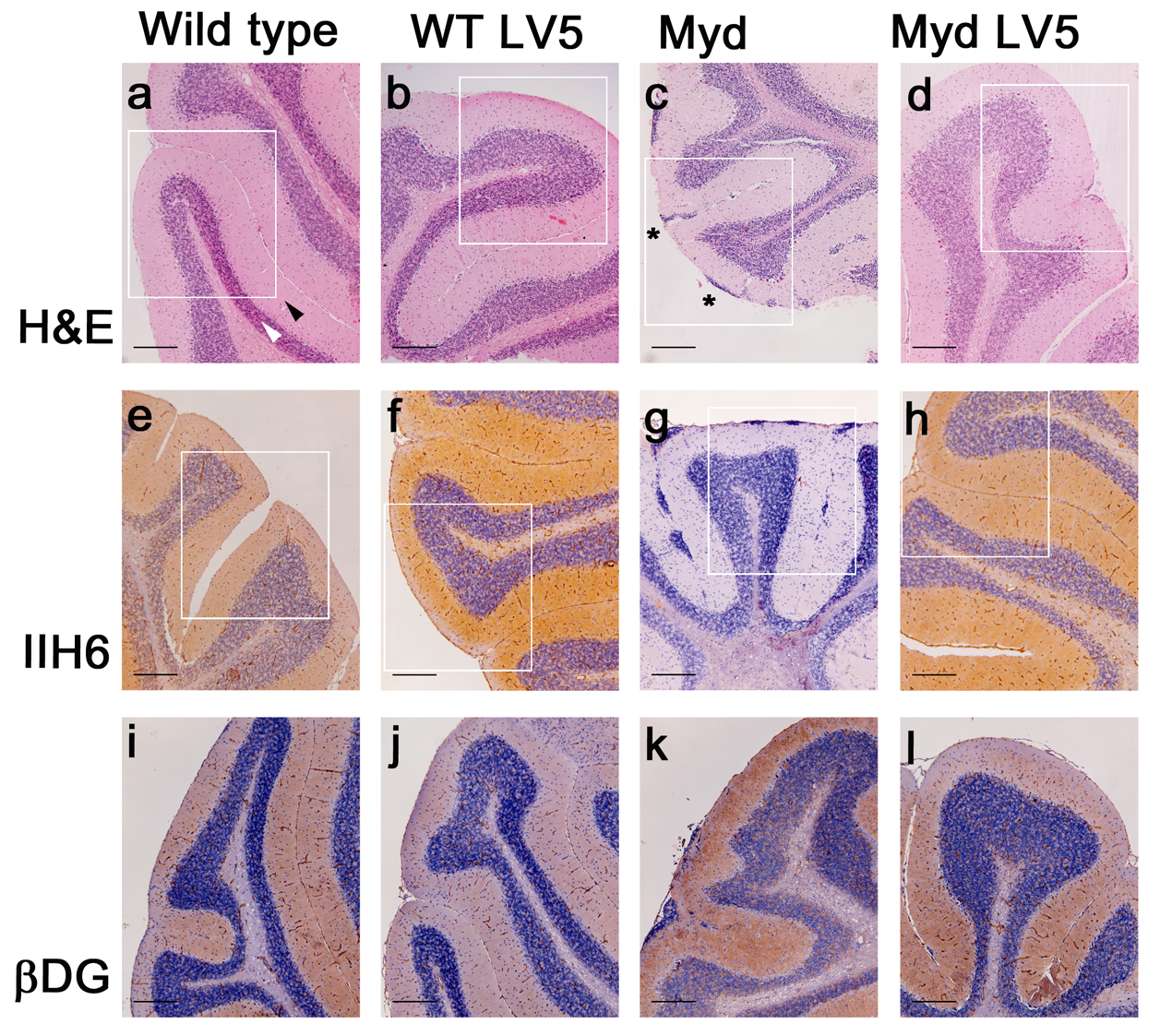

Supplement: S2 Fig — Cerebellar sections of brains from wild type, WT-LV5, LARGEmyd and LARGEmyd-LV5 mice (as indicated). a-d: Haematoxylin/Eosin staining. In WT and WT-LV5 mice (a, b), the molecular layer (Black arrowhead) and granular cell layer (White arrowhead) are readily apparent, with a single layer of large Purkinjie cells sandwiched between them. In LARGEmyd mice (c), the granular cell layer is extensively disrupted with large aggregates of ectopic granule cells superficial to the molecular layer (asterisks). This disruption is corrected, and ectopic granule foci greatly reduced, in the LARGEmyd-LV5 mice (d). e-h: IIH6 immunostaining. IIH6 reactivity is observed in blood vessels and the pia in WT, WT-LV5 and LARGEmyd-LV5 cerebellum, but not in brains of LARGEmyd mice. Overall stain intensity is higher in LV5 transgenic mice than in WT controls. i-l: β-DG immunostaining. Pia and blood vessels are visible in the cerebellum of WT, WT-LV5 and LARGEmyd-LV5 mice but not in LARGEmyd mice. LARGEmyd mice instead display a diffuse, indistinct staining of the molecular layer. Bars represent 200μm. White boxes: image subsections shown in Figs 5 and 6 (see main text). (TIF) [file pone.0159853.s002.tif]

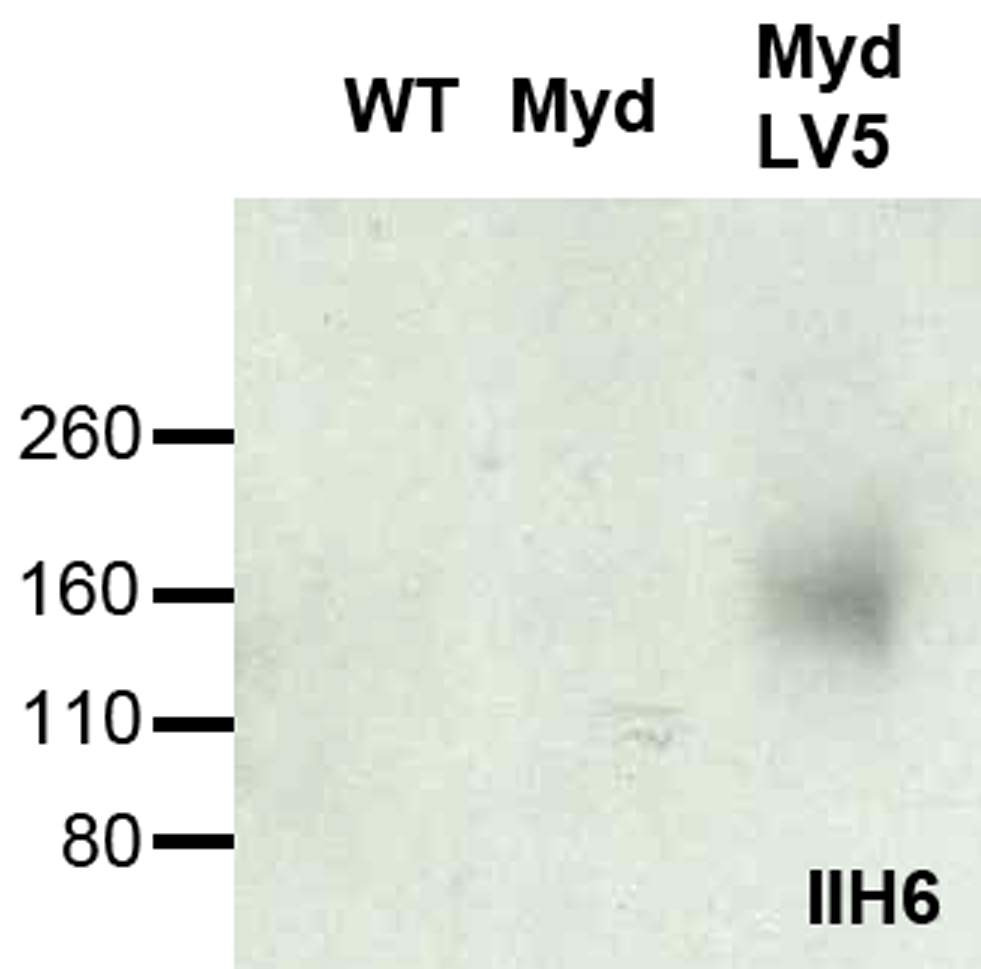

Supplement: S3 Fig — IIH6 western blot of tissue lysates from testis of WT, LARGEmyd and LARGEmyd-LV5 mice (as indicated). (TIF) [file pone.0159853.s003.tif]
