# Supplementary material for: Transgenic Rescue of the LARGEmyd Mouse: A LARGE Therapeutic Window?
Source: PLoS One. 2016 Jul 28;11(7):e0159853. doi: 10.1371/journal.pone.0159853 (PMC4965172; doi:10.1371/journal.pone.0159853)
Supplement: S1 Sequences — (DOC) [file pone.0159853.s004.doc]

**S1 Sequences: qPCR primers used for LARGE2**

mLarge2 F1 CCTGGATACCGACGTCACTT

mLarge2 R1 GGCTGTCACCTTCCACATCT

mLarge2 F3 TGGGCAGGGGATTTAACACA

mLarge2 R3 GTCTGACAGCTGCACGTTC

mLarge2 F4 GTCTCGTGGAGAACCAGAGC

mLarge2 R4 GACCGCATTGAAGATGTCCT

mLarge2 F5 GTTGGCTTCTTCCTGTTCGG

mLarge2 R5 GAGTTGTATCCCGCACACAC

mLarge2 F6 TGAACCCTACGTGGTGGTAC

mLarge2 R6 GTGGGGCAAGTGGATAGAGA
